# Mapping the Neural Substrates of Recent and Remote Visual Imprinting Memory in the Chick Brain

**DOI:** 10.3389/fphys.2019.00351

**Published:** 2019-03-29

**Authors:** Anna A. Tiunova, Natalia V. Komissarova, Konstantin V. Anokhin

**Affiliations:** ^1^Laboratory for Neurobiology of Memory, P.K. Anokhin Research Institute of Normal Physiology, Moscow, Russia; ^2^Department of Neuroscience, National Research Center “Kurchatov Institute”, Moscow, Russia; ^3^Institute for Advanced Brain Studies, Lomonosov Moscow State University, Moscow, Russia

**Keywords:** chicks, imprinting, learning, memory, retrieval, systems consolidation, *c-fos* expression

## Abstract

Social attachment formed by filial imprinting in newborn chicks undergoes a process of memory consolidation that involves rearrangement of its neural storage substrates. In the first 3 h after imprinting it depends on the integrity of the intermediate medial mesopallium (IMM) and beyond that time on unidentified memory storage structures dubbed S’. To search for the S’ memory system in the chick brain, we mapped and compared patterns of activity induced by retrieval of filial attachment memory before and after this critical transition. Chicks were trained in the visual imprinting task, and their memory was reactivated by imprinting stimulus either 1 h (recent memory retrieval) or 24 h (remote memory retrieval) after the completion of training. Patterns of brain activity were mapped by *in situ* hybridization to mRNA of an immediate early gene *c-fos*. We also mapped *c-fos* expression induced by the first presentation of the imprinting stimulus. Memory retrieval triggered massive *c-fos* expression in the chick brain both 1 and 24 h after the end of training. These activity patterns mostly coincided with the *c-fos* expression induced by the first presentation of imprinting stimulus. However, in the hippocampus *c-fos* induction was observed only after the first exposure to imprinting stimulus but not after memory retrieval. In the IMM, medio-rostral nidopallium/mesopallium, and hyperpallium densocellulare *c-fos* activation was induced by retrieval of only the remote but not of the recent memory. These *c-fos* mapping data point to the candidate brain structures for systems reorganization of imprinting memory in chicks.

## Introduction

Chicks of precocial birds form strong preference for a moving object that they encounter within the first hours of their life. In the brain of domestic chicks (*Gallus gallus domesticus*), visual imprinting depends on the intermediate medial mesopallium (IMM). Bilateral lesions of the IMM before training prevent learning and the lesions made less than 3 h after the training disrupt the acquired memory (McCabe et al., 1982). However, there is also an additional memory system (named S’) that does not depend on the IMM integrity. In contrast to the IMM-dependent memory, S’ system becomes functional 4–6 h after the end of training, and by 26 h it is fully able to sustain the imprinting recall in the absence of the IMM (Cipolla-Neto et al., 1982; Honey et al., 1995).

Despite the existence of S’ system was hypothesized long time ago, the neural substrate of this additional memory storage is still unknown. In the present study we addressed this question by comparing neuronal activation induced in the chick brain by the recent (1 h after the end of training) and by the remote (24 h) retrieval of imprinting memory. For this purpose, we used *in situ* hybridization mapping of stimulus-induced expression of an immediate early gene *c-fos* known to be regulated by neuronal activation (Minatohara et al., 2016) and expressed during formation and retrieval of memory in the chick brain (McCabe and Horn, 1994; Suge and McCabe, 2004; Salinska, 2006; Suge et al., 2010; Yamaguchi et al., 2010).

## Materials and Methods

Chicken embryos of the Ptichnoe strain were obtained from a local supplier on E12-E15 and incubated in darkness until hatching and imprinting (see Supplementary Figure 1 for the scheme of the experiment). At the age of 24 ± 8 h chicks were placed in a running wheel and exposed to an imprinting object (illuminated rotating box) for 60 min. Species-specific maternal calls were played back during the training. The number of the wheel revolutions toward the training stimulus and in the opposite direction was recorded. After the training chicks were returned to the home boxes and left until the memory retrieval session either 1 h after the end of training (Recent memory retrieval Group, *n* = 10) or 24 h after the training (Remote memory retrieval Group, *n* = 10). For memory retrieval, chicks were placed in the running wheel and exposed to the same imprinting stimulus for 20 min. Immediately afterward chicks were sacrificed, and their brains were processed for *in situ* hybridization. Additionally, there were 3 control groups. Chicks of the first exposure group [1st Exp (1st Exposure) Group] (*n* = 9) were placed in the running wheel and exposed to the imprinting object for 20 min without preceding training, their brains were taken for *in situ* hybridization immediately after this session. Chicks which received training without the retrieval session (Training Group, *n* = 9) were trained for 60 min and sacrificed 1 h 20 min later. The quiet control chicks (QC Group, *n* = 8) were kept individually in dark boxes and taken for *in situ* hybridization from there.

**FIGURE 1:**
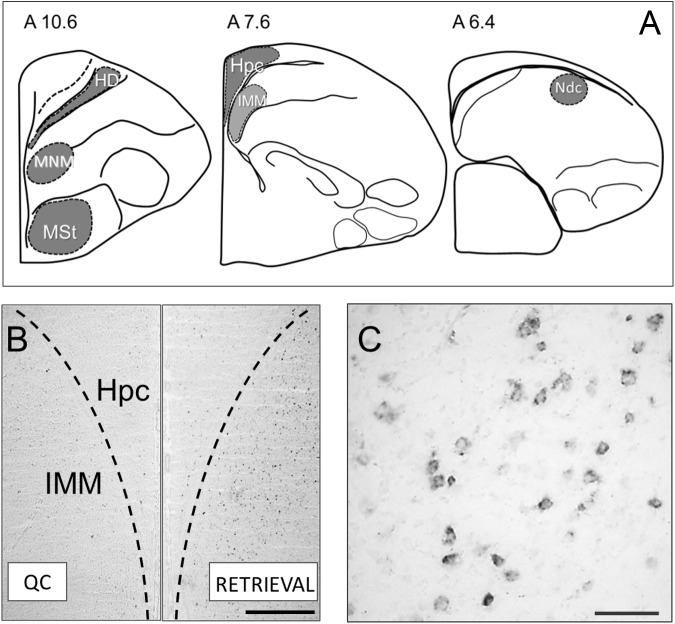
**(A)** Brain structures used for the *c-fos* expression analysis: HD, Hyperpallium densocellulare; MNM, Medio-rostral nidopallium/mesopallium (Maier and Scheich, 1983); MSt, Medial striatum; Hpc, Hippocampus; IMM, Intermediate medial mesopallium (Horn et al., 1983); Ndc, Nidopallium dorso-caudale (Metzger et al., 1998). **(B)** Micrographs showing representative *c-fos* mRNA staining in the brain of the untrained control chick (left half) and after memory retrieval (right half of the image). Scale bar = 500 μm. **(C)** Micrograph showing cytoplasmic *c-fos* mRNA staining at a higher magnification. Scale bar = 100 μm.

*C-fos* mRNA was detected by *in situ* hybridization on 20 μm cryostat brain sections with the 502 bp digoxigenin-labeled chicken *c-fos* RNA probe synthesized according to the manufacturer’s protocol (DIG RNA SP6/T7 Labeling Kit, Roche). The *c-fos* mRNA detection protocol was described elsewhere (Della Ragione et al., 2006). Sections were digitized and quantitative analysis was carried out in six brain regions – the intermediate medial mesopallium (IMM), medio-rostral nidopallium/mesopallium (MNM), medial striatum (MSt), hyperpallium densocellulare (HD), nidopallium dorsocaudal (Ndc), and the hippocampus (Hpc) (Horn et al., 1983; Kuenzel and Masson, 1988; Metzger et al., 1998; Suge and McCabe, 2004; Thode et al., 2005; see Figure 1A). Expression density was calculated as the ratio of the number of labeled cells in the selected region to the region area in mm^2^. Statistical analysis was carried out using Statistica 6.0. To meet ANOVA assumptions, the data were log-transformed and the between-group differences were estimated using one-way ANOVA. *Post hoc* analysis was performed using the Tukey HSD test.

This study was carried out in accordance with the recommendations of the Directive 2010/63/EU of the European Parliament and of the Council of the European Union issued September 22, 2010, on the protection of animals, used for scientific purposes (Section 27). The protocol was approved by the Ethics committee of the Anokhin Research Institute of Normal Physiology.

## Results

Mean number of the wheel revolutions during 20 min sessions was 91.2 ± 32.4 (mean ± SE) for the recent memory retrieval group and 113.0 ± 70.2 for the remote memory retrieval group, while for the first exposure group the mean number of revolutions was 16.6 ± 7.2 which was significantly less than in the retrieval groups [F(2,24) = 4.23, *p* = 0.028].

No significant interhemispheric differences were found in *c-fos* mRNA expression in all six analyzed brain regions, therefore the data from the left and right hemispheres were pooled. The ANOVA revealed significant between-group differences for the IMM [F(4,39) = 6.58, *p* = 0.00038], MNM [F(4,25) = 8.97, *p* = 0.00012] and HD [F(4,43) = 20.14, *p* = 0.00000]. Pronounced elevation of *c-fos* expression in these structures was observed in the remote memory retrieval group and in the first exposure group compared to the quiet control and to the training group which received no memory retrieval (Figure 1A–C).

The level of *c-fos* expression in the recent memory retrieval group did not differ from the quiet control group and from the training group (Figure 1A–C).

**FIGURE 2:**
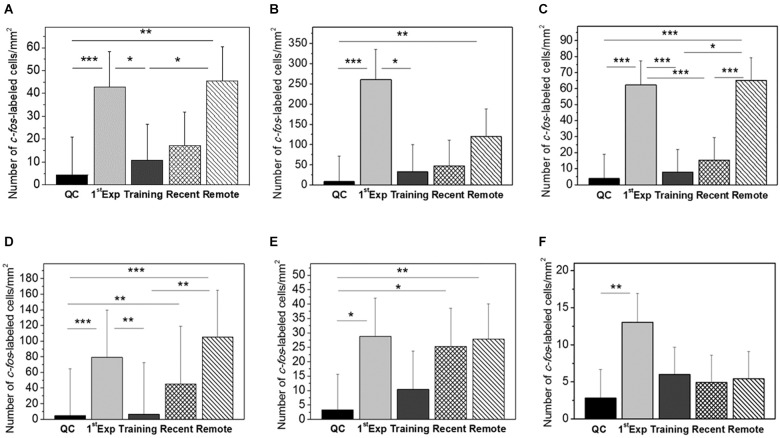
Mean number of *c-fos*-labeled cells in mm^2^ in the IMM **(A)**, MNM **(B)**, HD **(C)**, MSt **(D)**, Ndc **(E)**, and Hpc **(F)**. Groups: QC, dark-reared chicks taken from their homeboxes; 1st Exposure, chicks were exposed for 20 min to the imprinting object and the brains taken immediately after the session; Training, brains taken 1 h 20 min after 60-min exposure to the imprinting object; Recent, brains taken immediately after 20-min re-exposure to the imprinting object, the interval between the first (60-min) exposure and the re-exposure 60 min; Remote, brains taken immediately after 20-min re-exposure to the imprinting object, the interval between the first (60-min) exposure and the re-exposure 24 h. Error bars denote 0.95 confidence intervals. ^∗^*P* < 0.05; ^∗∗^*P* < 0.01; ^∗∗∗^*P* < 0.001. The between–groups differences were estimated using the one-way ANOVA followed by Tukey HSD applied to log-transformed data.

Significant group effect was found in the Ndc [F(4,26) = 6.03, *p* = 0.00143] and MSt [F(4,26) = 12.83, *p* = 0.00006] as well. In these areas both recent and remote memory retrieval induced *c-fos* expression comparable to that in chicks which were presented with the imprinting stimulus for the first time [1st Exp (1st Exposure)] (Figure 2D,E). In the hippocampus no induction was observed after the recent and remote memory retrieval while the first exposure to the imprinting stimulus induced strong expression [F(4,42) = 4.26, *p* = 0.00552, Figure 2F].

## Discussion

The aim of the present study was to identify structures of the chick brain that had differential activation during retrieval of the recent (1 h) and delayed (24 h) visual imprinting memory. In this analysis we relied on the known property of *c-fos* to be a marker of activity-dependent neuronal transcription (Minatohara et al., 2016). During re-exposure to imprinting object this activation may subserve memory reconsolidation (Litvin and Anokhin, 2000; Anokhin et al., 2002) and thus localize memory storage sites.

The weak *c-fos* expression in the quiet control chicks supports our previous observation on its low basal expression in the newborn chicks (Anokhin et al., 1991). The expression in the trained group 80 min after the end of training did not differ from the controls confirming a rapid decay of transiently induced *c-fos* mRNA (Figure 2). The highest level of *c-fos* expression was in chicks exposed to the imprinting object for 20 min [1st Exp (1st Exposure) Group]. These results are in line with the data that imprinting-induced *c-fos* expression in the IMM reaches maximum after the 15-min of training and returns to the basal level 75 min after the session completion (Suge et al., 2010).

We found that retrieval of imprinting memory 1 and 24 h after the training induced expression of *c-fos* in several brain regions. The density of *c-fos* positive cells was higher in the remote retrieval group compared with the recent retrieval group in most of the examined structures. After the remote retrieval, *c-fos* expression was significantly increased in the IMM, MNM, MSt, HD, and Ndc as compared with the quiet control group (Figure 1).

The expression of *c-fos* in the IMM was induced only by the remote but not the recent memory retrieval (Figure 2A). However, electrophysiological recording of the IMM neuronal responses to imprinting stimulus revealed two peaks of high responsiveness – at about 1.75 and 25 h after the onset of training (Horn et al., 2001). These intervals coincide with retrieval sessions in our experiments. Thus, a day after training IMM shows both electrophysiological and *c-fos* neuronal responses which supports the view that IMM participates in the retrieval of imprinting memory at 24 h after the training (Horn, 2004), while the engagement of IMM in retrieval of the recent memory is documented by the electrophysiological (Horn et al., 2001) and the lesion data (McCabe et al., 1982). The dissociation between electrophysiological and *c-fos* data can be due to different aspects of neuronal functions measured by two techniques, *c-fos* being preferentially a plasticity marker.

A similar pattern of differential *c-fos* expression in the recent and remote retrieval was observed in the MNM and HD (Figure 2B,C). MNM was defined by learning-induced increase in 2-deoxy-D-glucose uptake, release of glutamate and expression of another immediate early gene ZENK during acoustic imprinting (Maier and Scheich, 1983; Gruss and Braun, 1996; Bredenkötter and Braun, 1997; Thode et al., 2005). HD core connects the Wulst and IMM and this link is strengthened by imprinting (Nakamori et al., 2013). Selective lesions of the HD impair imprinting (Nakamori et al., 2010). Moreover, c-Fos expression in the HD neurons was activated by the presentation of imprinting stimulus to the P7 day chicks imprinted on the P1 (Nakamori et al., 2010). Our results on the remote retrieval-induced *c-fos* HD expression are in line with these data.

In the MSt and Ndc the *c-fos* expression was increased by both recent and remote memory retrieval (Figure 2D,E). Ndc is a nidopallium area discovered by the increased metabolic activity during presentation of the imprinting stimulus to the acoustically or visually imprinted chicks (Bock et al., 1997). 30 min of acoustic imprinting induced expression of the *Arc* gene in the Ndc (Bock et al., 2005), and there was a reduction in spine density in this area after imprinting (Braun et al., 1999). Blockade of NMDA receptors in the Ndc impaired imprinting (Bock et al., 1997). Since Ndc projects to the IMM and is reciprocally connected with the MNM it was suggested that Ndc represents an associative brain region integrating visual and acoustic features of imprinting objects (Braun et al., 1999). MSt belong to the basal ganglia system important for learning and memory in chicks (Gilbert et al., 1991; Csillag, 1999), though kainate lesions of MSt were without effects on chick approaching behavior in the imprinting test (Izawa et al., 2001), which calls for cautionary interpretation of *c-fos* imaging data alone.

Finally, in the hippocampus *c-fos* expression was induced by the first exposure of chicks to the imprinting stimulus but not by the retrieval of imprinting memory (Figure 2F). The hippocampus in the chick projects bilaterally to the IMM (Bradley et al., 1985). However, 24 h after imprinting hippocampal neurons were shown to be sensitive only to the distance to the imprinting object but not to the specific object’s characteristics (Nicol et al., 1998). Also, 15 min of imprinting training induced c-Fos expression in the hippocampus, but the level of the expression did not correlate with the preference score (Suge and McCabe, 2004). Our data support the view that the hippocampus is recruited during acquisition but not the retrieval of the imprinting memory.

In general, our study revealed a number of brain structures that were activated by the recent and remote retrieval of imprinting memory. They also show that the 24 h memory retrieval induced a broader *c-fos* expression than the retrieval of the 1 h memory. It was previously hypothesized that by the 24 h two parallel systems are supporting the imprinting memory, IMM-based system and the S’ system with unknown location (Horn et al., 2001; Horn, 2004). Our *c-fos* mapping data suggest several brain regions which may represent the S’ system. Other candidate structures will need to be examined as well, particularly the intermediate hyperpallium apicale which receives direct neural projections from the IMM and plays a critical role in imprinting retention and recall in chicks (Aoki et al., 2015). As data on the MSt indicate (Izawa et al., 2001) lesion experiments are required to test the role of mapping-identified structures in storage and retrieval of filial attachment memory.

## Data Availability

The raw data supporting the conclusions of this manuscript will be made available by the authors, without undue reservation, to any qualified researcher.

## Author Contributions

KA and AT conceived the study and wrote the manuscript. AT and NK designed and performed the experiments and analyzed the data.

## Conflict of Interest Statement

The authors declare that the research was conducted in the absence of any commercial or financial relationships that could be construed as a potential conflict of interest.
