# Supplementary material for: Mapping the Neural Substrates of Recent and Remote Visual Imprinting Memory in the Chick Brain
Source: Front Physiol. 2019 Mar 29;10:351. doi: 10.3389/fphys.2019.00351 (PMC6450189; doi:10.3389/fphys.2019.00351)
Supplement: Supplementary file 1 [file Table_1.DOCX]

**Supplementary Figures**


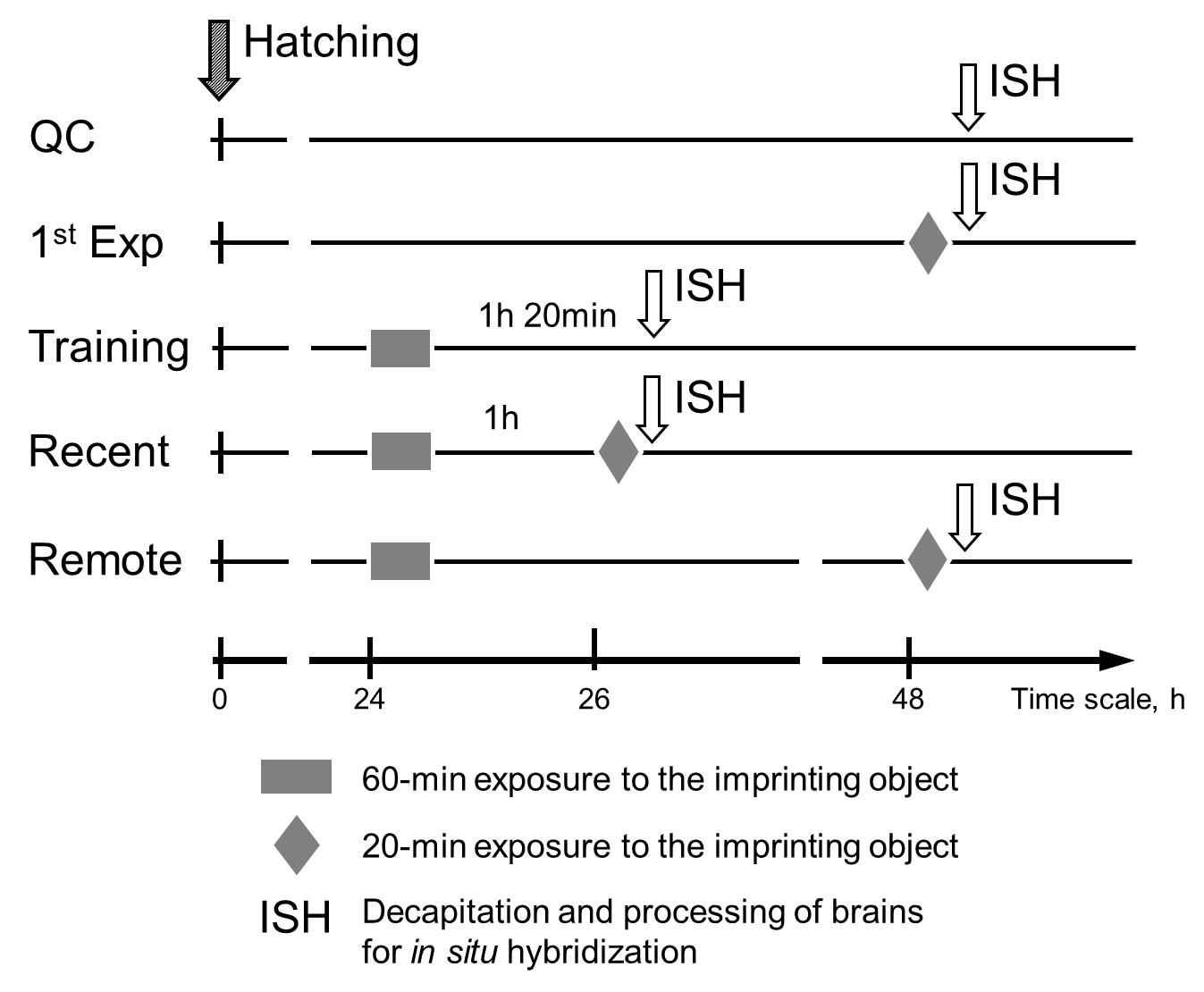


**Supplementary Figure 1.** Experimental groups and the design of the experiment (see also Materials and Methods).
